# Patient Reported Outcome Measurement (PROM) under real-life conditions of non-curable cancer outpatients with the Integrated Palliative Outcome Scale (IPOS) and NCCN-Distress Thermometer – A mixed methods study

**DOI:** 10.1016/j.pecinn.2024.100264

**Published:** 2024-02-12

**Authors:** Eileen Ratzel, Ina Maria Pretzell, Thomas Kindler, Martin Weber, Christina Gerlach

**Affiliations:** a Interdisciplinary Department of Palliative Care, III. Department of Medicine, University Medical Center of the Johannes Gutenberg University of Mainz, Langenbeckstraße 1, Geb. 407, 55131 Mainz, Germany; b University Cancer Center Mainz (UCT Mainz), University Medical Center of the Johannes Gutenberg-University of Mainz, Germany; c Department of Palliative Care, Heidelberg University Hospital, Im Neuenheimer Feld 305, 69120 Heidelberg, Germany

**Keywords:** Palliative care, Screening, Integrated palliative care outcome scale (IPOS), Distress thermometer, Quality of life, Patient reported outcome measurement

## Abstract

**Objective:**

Prospective cohort study to test the real-life feasibility of longitudinal patient-reported outcome measurement PROM (Integrated Palliative Outcome Scale IPOS, and NCCN Distress Thermometer DT) required for outpatients with non-curable lung or prostate cancer in comprehensive cancer centers.

**Methods:**

Assessment with paper-based IPOS and DT was observed for 15 months. We analyzed response to patients' distress (requests for supportive and palliative services) following PROM. Focus groups to comprehensively explore the user experience of patients, informal caregivers and health care professionals (HCP) supplemented the analysis.

**Results:**

Ninety-seven percent (125/129) of the patients received a questionnaire once, but quarterly assessment as recommended by certification committees was achieved only in 50% and 31% of prostate and lung cancer patients. Although both instruments were well accepted, only IPOS showed a high content validity, because some patients had difficulties in understanding the DT. Patients felt comfortable with completing the PROM, and HCP found PROM helped to structure the patient encounter. Due to organizational deficiencies in the handling of the instruments and operationalization of reactions to identified distress, the referrals to supportive and palliative services were rare.

**Conclusion:**

To facilitate consequences from PROM it should be a standardized intervention rather than assessment alone.

**Innovation:**

The patient perspective improves the implementation of PROM under real-life clinical conditions.

## Highlights

• implementation of PROM under real-life clinical conditions improved by patient perspective.
• doctors sensitized by PROM to distress of patients, who appreciate responses.
• IPOS preferred by patients.

## 1 Introduction

About 50% of patients with lung cancer and 20% with prostate cancer are diagnosed in advanced stages [[1], [2], [3]], which are associated with symptoms and psychosocial burden [[4], [5], [6], [31]]. Quality of life (QoL) is, therefore, a major treatment goal in palliative situations. Thus, to improve patients' quality of life, the German Cancer Society (DKG) requires that oncology centers seeking certification use Patient Reported Outcome Measurements (PROM) [[7], [8], [9]]. PROM is a generic term for different concepts for measuring the subjectively perceived state of health without objective evaluation by outsiders. The information from PROM reflects the patient's subjective view of the illness experience and the influence of an illness on an individual's life [10].

PROM has been shown to be effective in stabilizing and improving cancer patients' QoL, preventing emergencies, helping to remain on tumor-specific therapies, and to improve survival while no adverse effects are known from such assessments [11,12]. The benefit of an established PROM based screening tool for distress, the distress thermometer DT, has been shown in previous studies in patients with advanced lung and prostate cancer [[13], [14], [15]]. However, due to its purpose for screening, the design of the thermometer does not allow for a differentiated assessment of patients' distress. The patients indicate their general distress level on the thermometer figure. In a second step they mark the presence and absence of a problem in a problem list, but not whether and how stressful each individual problem is perceived by the patient. The DT problem list could serve as a trigger for referrals to supportive services [16], but it is not validated [17]. In contrast, IPOS (Integrated Palliative care Outcome Scale) was proven to be a useful PROM tool to assess patient distress and QoL in detail [18]. It was developed with the involvement of patients and uses a Likert scale to assess the subjective burden caused by the respective problem. IPOS is validated for clinical routine care as well as research; its sensitivity for unmet palliative care needs has been confirmed [19,20]. We wanted to observe whether one of the two instruments has advantages over the other in everyday clinical practice in terms of user friendliness, both in patient comfort and ease of interpretation.

We systematically analyzed the feasibility of PROM with DT and IPOS in the clinical routine care of vulnerable oncology outpatients. In addition, by using both questionnaires simultaneously, a direct comparison was possible. Enhancing and inhibiting factors of meeting audit demands were explored in focus group interviews with patients, informal caregivers (ICG) and health care professionals (HCP). Thus, a prospective cohort study design with integrated qualitative analysis was chosen. The results aim to facilitate sustainable individualized interdisciplinary care using the full range of supportive and palliative care services.

## 2 Methods

The study follows the ‘STrengthening the Reporting of OBservational studies in Epidemiology’ (STROBE) [21], ‘COnsolidated criteria of REporting Qualitative research’ reporting guidelines [22], and Good Reporting of A Mixed Methods Study (GRAMMS) [23] (Supplement 1).

### 2.1 Sample and setting

The setting was the outpatient clinic of a comprehensive cancer center receiving an average of 786 patients per quarter situated at a university hospital (tertiary center) in Germany. It was running for 8 h on weekdays with a reception staffed with 2–3 medical assistants, diagnostics and 10 treatment stations staffed by 3–4 nurses, and doctors from various oncological disciplines in 4 medical consultation rooms. Patients with non-curable lung cancer or castration-resistant prostate cancer (CRPC), their ICGs, as well as physicians and nurses of the outpatient clinic were included. Exclusion criteria for the quantitative and the qualitative part of the study were age < 18 years, and insufficient language skills to fill in the German-language questionnaires. For ethical reasons additional exclusion criteria for the qualitative study part were a KPS (Karnofsky Performance Status) of 50% or less, severe stress or cognitive impairment, and a life expectancy of a few days as assessed by the treating team. Prostate cancer was chosen for the study because of the new DKG certification requirement for PROM, and lung cancer because of the high number of patients known to need palliative care, and evidence from studies for the effectiveness of early integration of palliative care in this patient group [5,[24], [30]].

### 2.2 Data collection

The study consists of a quantitative and a qualitative part. We observed delivery and return of DT and IPOS to the eligible patients for 15 months, the identified distress requiring consultation of the patient and/or referral to social service, psycho-oncology, pain and palliative care consultation, the respective HCP reaction, and we explored the experience of patients, relatives, and HCP with the PROM in this setting in focus groups. The comprehensive cancer center daily lists in the clinical information system were used to find out how many eligible patients actually visited the outpatient clinic. We informed all staff members at the beginning about the PROM-based screening and trained new employees as the study progressed. In addition, we supported the work-flow with written material and process charts and provided information at the monthly outpatient department meetings.

### 2.3 Quantitative methods

The DT had already been established for routine clinical assessment, but implementation and results had not been evaluated. Outpatient clinic's physicians were asked to identify eligible patients and note on their routing slips to provide them with an IPOS in addition to the DT at their next appointment, and the patients' charts were labeled with a sticker “IPOS”. The distribution of questionnaires was handled by nursing staff at a patient's first appointment in the respective quarter of the year. The process was supported by a research assistant in the 5th quarter of the study to check for confounders, e.g. gate keeping, time constraints, inadvertent forgetting. Patients filled in the paper-based PROM while waiting and were asked to hand them to their attending physicians at consultation.

#### 2.3.1 National Comprehensive Cancer Network's Distress Thermometer (NCCN-DT)

The instrument is a free resource. In the current original format, problem list and thermometer appear next to each other on a broadsheet (https://www.nccn.org/docs/default-source/patient-resources/nccn_distress_thermometer.pdf). The dichotomous problem list can be used to identify the origin of a patient's distress, however, the German version of the problem list (V.1.2016) has yet to be validated [17,25], and had been empirically modified by the University Medical Center Mainz, Department of Psycho-oncology using a portrait format (Supplement 2). Increased distress was defined as a measure of five or higher on the thermometer [25]. The attending physicians were asked to indicate whether they referred their patients to a supportive or palliative care service, and to sign the questionnaires.

#### 2.3.2 Integrated Palliative Outcome Scale (IPOS)

Use of the IPOS is free of charge, but registration on the website https://pos-pal.org is required. The German one-week recall patient version of the IPOS [18] consists of two pages with 17 questions with Likert-Scale (0–4) answer options with higher values indicating greater subjective stress. IPOS starts with a free-text field where patients can describe what problems and concerns affected them during the past week. The second question is a symptom list, which can be continued by the patient with additional symptoms experienced in the past week. Further questions refer to psycho-social burden, and the need for additional social and prognostic counselling. If one item of IPOS was marked three or four, increased distress was indicated. Attending physicians were asked to sign the questionnaires and indicate whether they could solve the patient's problems by consultation only, or if they referred the patient to social service, psycho-oncology, pain clinic, and/or palliative care (Supplement 2).

### 2.4 Data analysis

#### 2.4.1 Statistics

A binomial test [26] whether the frequency distribution of a dichotomous variable corresponds to an assumed distribution at a significance level of 5% was used to check if the DKG's specifications for questionnaire distribution (100% of eligible patients received a questionnaire) were met [8].

Independence of variables in fourfold tables was calculated with Pearson's chi-squared tests, and Fisher's exact test was computed with figures below five [27], using SPSS 23 to analyze whether a referral to supportive and palliative services or a counselling had taken place following a positive screening result for increased distress, or by chance.

### 2.5 Qualitative methods

Field notes to record observations were constantly taken during the project.

Focus group participants were identified by purposive sampling. The research assistant invited in person and by e-mail all HCP involved in the screening process to take part in a focus group exploring their experiences and opinions regarding the screening. To avoid additional travels patients with an appointment at the respective focus group dates and their ICG were in advance invited in person or by call. All participants provided informed consent and were given the option to fill in a demographic data sheet. Focus group interviews with patients, their ICG and HCPs were based on topic guides developed by the authors (Supplement 3). For all three participant groups we included open questions about the organization and process of PROM, the personal opinion and experience with the two instruments. Additionally, we provided the opportunity to patients and HCP to report about the impact of cancer on their everyday quality of life. The interviews were conducted by C.G., a trained clinical scientist (M.Sc.) and physician, audio-recorded and transcribed verbatim (anonymized). The transcripts were thematically analyzed using the framework approach [28,29], which is based on a matrix that helps to inductively organize the text and identify thematic categories from codes within and across the focus groups. The coders had different levels of prior experience, the first author E.R. was an undergraduate medical student pursuing her doctoral thesis and supported by C.G. Meaning sections were coded without counts of frequencies. Categories were identified from codes and condensed following iterative consultation and agreement of the coders. The inter-coder reliability correlation kappa was substantial for the patient focus groups (kappa 0.7 and 0.6, 0.6 and 0.5, 0.7 and 0.7 for structure, process, and outcome in the lung cancer and the CRPC group) [45]. Inconsistencies between coders were reconsidered and alternative interpretations incorporated into the analysis. M.W. (physician) and a psychologist, not involved in the study, would give advice in case of disagreement. A data matrix generated on the basis of identified categories and corresponding quotes was used as a template for the framework. Categories were further analyzed, emphasizing similarities and differences. In conclusion, comparisons were made, hypothesized, and explanations drafted.

### 2.6 Ethics approval and study registration

The ethics committee of Rhineland-Palatinate's General Medical Council approved the study (24-08-2017/26-04-2018, No.2018–13,219), and determined that no informed consent was required for the quantitative part of the study. All participants of the focus groups provided informed consent.

The study was registered in the clinic's document management system at the University Medical Center Mainz (UCT#1332), the FoR.UM project data base (no.20–03265), and with the German Clinical Trials Register (DRKS00023195).

## 3 Results

### 3.1 Distribution of PROM instruments

The charts of 103 eligible patients with advanced lung cancer (46 female; 89 with non-small cell lung cancer NSCLC, 14 with small cell lung cancer SCLC) and 26 patients with CRPC were regularly checked for PROMs during the study period of 15 months (01/04/2017–30/06/2018). Table 1a shows the demographic data.

**Table 1a:** Demographic data of patients who were supposed to receive PROM.

	Lung Cancer	CRPC
	n	n
**Sex**		
male	46	26
female	57	–

**Age (years)**		
30–39	0	0
40–49	6	0
50–59	25	0
60–69	37	10
70–79	30	11
80–89	5	5

**Entitiy**		
NSCLC	89	–
SCLC	14	–

CRPC = castration refractory prostate cancer,

NSCLC = non-small cell lung cancer, SCLC = small cell lung cancer.

**Fig. 1:**
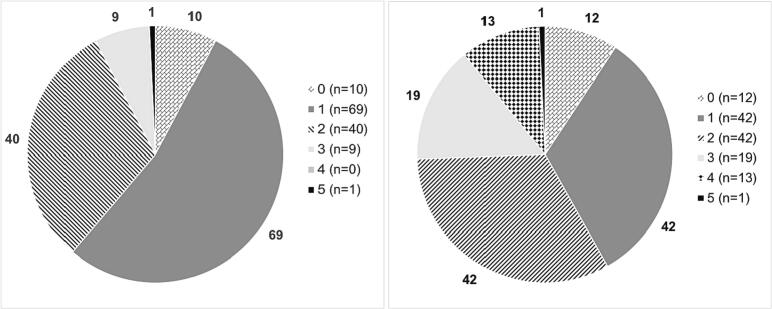
Number of PROMs (left: DT, right: IPOS) distributed to both patient groups during the five quarters (15 months) study period. Target value: one PROM with both instruments for each quarter the patient presented to the outpatient clinic.

Two-hundred-forty DT were distributed among the 129 patients, and 181 IPOS were distributed among 119 patients. Nearly all patients, 92 lung cancer patients (90%), and 25 (96%) patients with CRPC received at least one DT during the study period. The number of screenings of CRPC patients met the criteria of DKG to screen all patients, but lung cancer patients' screening was significantly lower (*p* < 0.001). Ninety-four lung cancer patients (91%) and 25 patients with CRPC (96%) received an IPOS assessment. However, quarterly screening for five quarters was only performed for one patient with DT, and for another patient with IPOS due to the varying numbers of patients to receive PROMs over the course of the study period (Table 3). Nevertheless, the number of actually distributed PROMs during the five quarters varied (Fig. 1).

When PROMs were distributed by a research assistant, screening rates increased considerably by an average of 49% (Table 3), but not the proportion of responses to distress.

Actually, 50% and 31% of prostate and lung cancer patients respectively received one of the PROMs as recommended, once every visiting quarter. Reasons for missing follow up were explored in qualitative interviews.

### 3.2 Inconsistency of identified signals and reaction to distress

**Fig. 2:**
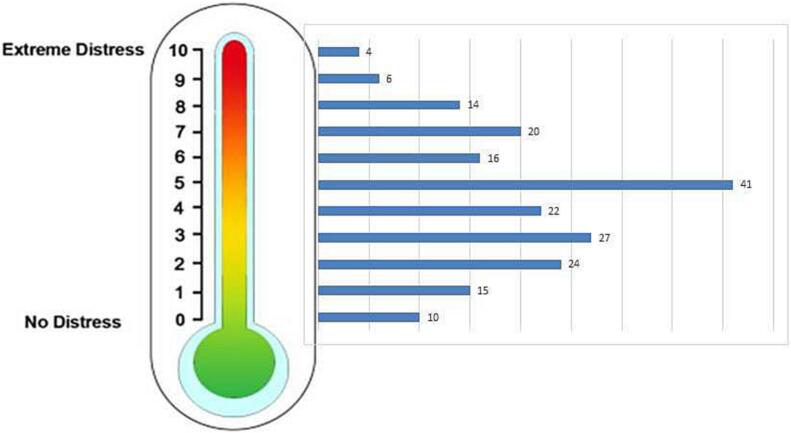
Distribution of values stated in NCCN-DT by advanced lung and prostate cancer patients.

Signals for distress occurred in 45% (86/190) of DTs in lung cancer patients, and in 32% (16/50) of patients with CRPC (Table 2; Fig. 2). However, none of the signals of distress indicated by the DT led to a consultation with supportive and palliative services for patients with CRPC. On 10/190 DT questionnaires (5%) physicians stated the enrollment of lung cancer patients with supportive and palliative services (all psycho-oncology), although four out of these 10 DT indicated no distress. Overall, distress indicated by DT did not correlate with referral to supportive and palliative services (exact Fisher *p* = 0.150).

IPOS of lung cancer patients indicated distress in 84/142 (59%) which was addressed in 49/84 (58%) cases (Table 2). Physicians solved patients' problems through consultation in 45 cases, and four patients were referred to supportive and palliative services (psycho-oncology for three patients, and pain and palliative care consultation for one patient). Nevertheless, in 17 cases (29%) of applying IPOS without any signal of distress, physicians identified patient distress they could mitigate through consultation. Lung cancer patients' distress indicated by IPOS was correlated with a reaction to problems, either by consultation by the attending physician or referral to supportive and palliative services (*p* = 0.001). An anonymized query in the clinical information system supported this result in the IPOS screened lung cancer patients with activity of social service, psycho-oncology, and palliative care in 19, 18, and 5 patients.

Compared to DT, IPOS indicated distress twice as much in CRPC, in 25/39 (64%) of cases, triggering reactions in 16/25 (64%), 15 consultations with the attending physician and one referral to supportive and palliative services, which was palliative care. However, in 8/14 cases (57%) of IPOS without any signal of distress physicians identified patient distress they solved by consultation (7/8) or referral to supportive and palliative services (1/8), which was palliative care again. Statistically, CRPC patients' distress indicated by IPOS was not correlated with a mitigating response (*p* = 0.673). Interestingly, the process of assessment for distress with IPOS led to supportive conversations; this may reflect a sensitization or other change of approach to patient problems, which was further explored in the focus groups.

### 3.3 The PROM experience

We conducted focus groups because observing whether the number of questionnaires distributed matches the certification requirements does not reveal the causes of deviations. The same applies to understanding the responses to distress identified during the screening process. We conducted focus groups with patients, ICGs and HCPs to explore their experience with DT and IPOS under everyday clinical conditions and to identify facilitators and obstacles for the use of PROM based screening that explain the falling below the screening requirements, the differences between the instruments to identify distress, and the response to distress.

The aim of the focus groups was to capture a 360° view on the PROM-based screening from all three populations. In the presentation of the results we have summarized the different perspectives thematically.

The total of six focus group interviews included 7 lung cancer and three CRPC patients, 9 HCPs (two medical assistants, four nurses, three physicians split in two mixed groups for organizational reasons) and three ICGs (two wives, one sister-in-law) split in two groups), reflecting a representative sample of those involved in PROM (Table 1b).

**Table 1b:** Participant demography of the six focus group interviews.

	**Lung Ca. Pts.**	**CRPC Pts.**	**HCP**	**ICG**
	**n**	**n**	**n**	**n**
**Total**	7	3	9	3

**Age (years)**				
30–39	0	0	7	0
50–59	3	0	2	0
60–69	1	3	0	1
70–79	1	0	0	2
n/s	2			

**Sex**				
female	2	0	7	3
male	5	3	2	0

**Martial Status**				
single	1	0	2	0
married/partnership	4	3	4	3
divorced	0	0	2	0
n/s.	2	0	1	0

**Educational level**				
Secundary education	1	0	5	3
Dual vocational training	1	0	1	0
University degree	2	0	1	0
PhD	0	0	2	0
n/s	3	0	0	0

**Religious affiliation**				
Roman-Catholic	1	1	5	2
Protestant	0	2	1	0
none	4	0	3	1
n/s	2			

**Migration background**				
Yes	1	0	3	0

**Role**				
Spouse	–	–	–	2
Other informal caregiver	–	–	–	1
Medical assistant	–	–	2	–
Nurse	–	–	4	–
Resident	–	–	2	–
Specialist	–	–	1	–

CRPC = castration refractory prostate cancer, HCP = healthcare professional,

ICG = informal caregiver, n/s = not specified, Pts. = patients.

**Table 2:** Distributed questionnaires, detected distress, and triggered response to PROM over five quarters (15 months).

	**DT**	**IPOS**
**Lung Cancer Patients**	190	142
Distress	86 (45%)	84 (59%)
Response to distress	6 (7%)	49 (58%)
Response without distress	4 (4%)	17 (29%)
**CRPC Patients**	50	39
Distress	16 (32%)	25 (64%)
Response to distress	0 (0%)	16 (64%)
Response without distress	0 (0%)	8 (57%)

CRPC = castration refractory prostate cancer.

**Table 3:** Eligible outpatients for PROM and actually distributed instruments.

	Quarter 1	Quarter 2	Quarter 3	Quarter 4	Quarter 5 monitored distribution	Increase in Q5 a
Eligible LC pts	66	69	70	65	76	
Distributed IPOS LC	46 (70%)	14 (20%)	14 (20%)	4 (6%)	64 (84%)	+ 55%
Distributed DT LC	43 (65%)	29 (42%)	28 (40%)	23 (35%)	67 (88%)	+ 43%
Eligible CRPC pts	18	18	16	15	15	
Distributed IPOS CRPC	9 (50%)	7 (39%)	4 (25%)	5 (33%)	14 (93%)	+ 56%
Distributed DT CRPC	10 (56%)	11 (61%)	10 (63%)	5 (33%)	14 (93%)	+ 40%

CRPC = castration refractory prostate cancer, DT = NCCN Distress Thermometer,

IPOS = Integrated Palliative Outcome Scale.

a Increase of distributed PROM instruments from mean of Q1–4 compared to Q5,

The focus groups took place in the fifth quarter of the study. The HCP-interviews took 31 and 23 min respectively, and involved 7 participants.

The focus group with 7 advanced lung cancer patients took 49 min. Three patients with CRPC were part of the second patient focus group, which took 33 min.

All participants of the ICG interviews were female. Their interviews took 10–30 min.

**Fig. 3:**
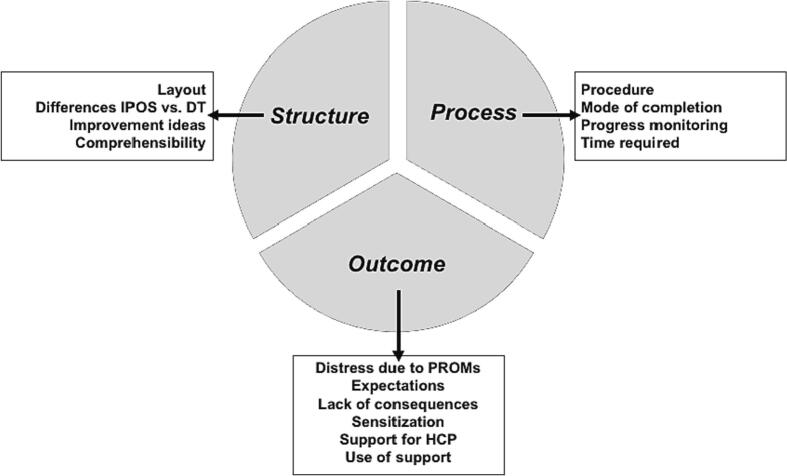
Figurative representation of the three categories and 14 subcategories related to PROM.

We identified three categories (structure, process, outcome) and 14 subcategories directly related to PROM (Fig. 3).

#### 3.3.1 Structure of PROM-based screening: layout, comprehensibility, and differences between IPOS and DT

HCPs and patients reported several times that it was more difficult to fill in the DT than the IPOS, because the thermometer-picture was sometimes misinterpreted, a confusing layout being a possible reason, or that the patient perception does not match the recommended cut off of five, which could explain part of the lack of response from doctors to positive screening.

*“Someone also confused up and down recently. That it was going super well, and ticked nine.”*

*(male doctor 2, second focus group)*

*“I do it like this, when I sleep well, I'm already happy with seven or six, yes, I'm perfectly happy.”*

*(patient 2, prostate cancer patient group)*

However, the single page layout was rated positively by most HCPs, but some criticized the DT being overstuffed. In contrast, patients and ICGs preferred the IPOS, because the Likert-Scale allows for grading of items, and some HCPs shared this opinion.

*“IPOS doesn't have yes or no answers. You can evaluate: did pain bother me a lot or less? This makes you reflect a little bit about how you really felt in recent weeks.”*

*(female nurse 3, first HCP focus group)*

The perceptions of ICGs and patients were actually in common. They preferred the IPOS, because they found it more sensitive.

*“Actually they [IPOS items] are written very, hm, precisely and very understandingly.”*

*(wife 2, ICG focus group)*

This experience was in contrast to some HCP, who perceived the IPOS wording too excessive. These physicians may be distracted from the IPOS results by its text, another possible explanation for the observed lack of responses to some patient distress.

*“I prefer the distress thermometer simply because it's only one page and, anyway, the information is more condensed. While the other one [IPOS] of course also contains.*

*a lot, a lot of poetry, which of course you can't necessarily read through every time.”*

*(doctor 1, first ICG focus group)*

All participants from the three populations appreciated the free-text option of the IPOS.

#### 3.3.2 Improvement ideas from the focus groups

Apart from several criticisms, the participants always mentioned ideas for improvement on how to better integrate PROMs in the clinical routine. HCP proposed to combine the DT picture with the IPOS items in one sheet. One patient thought of a short version because physical symptoms may change faster than psycho-social processes.

Patients and ICG felt they did not receive enough information due to insufficient communication but showed appreciation for time being a scarce resource.

*“Well, the questionnaires only make sense if they are evaluated. Basically … um … in terms of the questions, they are only useful if I [meaning the physician] can then respond to the patient.“.*

*(female patient 1, lung cancer focus group)*

They suggested drawing up an information sheet for patients, informing them about organizational processes relevant for all patients in the outpatient clinic, including concrete information about PROMs and how to use them.

#### 3.3.3 The process of PROM-based screening

This category summarizes the way a patient's mindset, the process of distributing PROMs, the processes in the outpatient clinic and the hospital in general influence screenings.

#### 3.3.4 Mode of completion, patients' expectations and mode of response

PROM is appreciated as a simple instrument, filled in swiftly by patients (1–10 min) to monitor symptom burden and QoL over time, which allows physicians to intervene in case of deterioration.

*“The doctor will be able to see at a glance when something has changed, if he gets this piece of paper at every consultation, and if he has the previous ones next to them.”*

*(female patient 1, lung cancer patient focus group)*

Patient appreciated when their doctors revisited problems that were mentioned in previous consultations, and the doctors found it advantageous that the questionnaires made it possible to maintain an overview of the patient's situation, even if they were seen by several colleagues.

However, they also noticed weaknesses in the process.

*“But I am afraid how they deal with the questionnaires depends entirely on their mood on the day.”*

*(female patient, lung cancer focus group)*

#### 3.3.5 Obstacles in the procedure

HCP critically reflected their omissions, neglecting to sign the PROM or to register consults as a direct response to distress. They also tolerated rather than corrected confused processes although these impeded the response process to distress and the respective consultation rates.

*“Sometimes they [patients] have already handed it [questionnaires] in at the front desk. Then it's stuck on the file. Sometimes they bring it in with them. Sometimes they have it in their jumble, forget to hand it in and then come back and hand it in again. Or in between they pull it out. It varies.”*

*(doctor 2, second HCP focus group]*

The lack of routine in distributing the questionnaires led to it being forgotten, which was investigated during the fifth quarter when E.R. was in charge. Also, stressful situations caused nurses to make mistakes, forgetting the indication stickers for IPOS at the patients' charts or producing IPOS sheets with the same print on both pages.

*„One reason could be when there was a rush at admission because many patients came in at the same time, the stickers on the patient charts were simply overlooked “*

*(female nurse 1, first HCP focus group)*

Furthermore, one staff member was afraid of burdening patients and ignored the IPOS.

*„As I said before, I don't like distributing it. I think when somebody comes with a tentative diagnosis I can't give him this piece of paper. They'll flip, I'd say, at least I would.”*

*(female nurse 2, first HCP focus group)*

However, this was not verified by patients who were asked about their distress caused by DT and IPOS itself.

*„This is part of …ah …good diagnostics, I'd say.“*

*(male patient 3, prostate cancer focus group)*

Nevertheless, reluctance of some HCP to hand out the PROMs explains beyond organizational issues the insufficient screening rates. This barrier could be overcome with staff education about the coping of patients with PROM and the harm caused by unmet need.

Eventually, the participants proposed to provide access to PROM based screening to all patients, not only the patients with incurable CRPC or lung cancers. This approach would simplify the distribution process and improve the care for patients with unmet needs.

*“Potentially, every oncology patient is a candidate who should be screened. Simply so that certain points are not overlooked.”*

*(medical assistant 1, first HCP focus group)*

#### 3.3.6 Outcome: Impact of the PROM-based screening

In this context, patients reported their expectations of noticeable consequences from PROM results, otherwise they felt frustrated. Those referred to supportive and palliative services consistently reported a positive experience and recommended them to other participants during the focus groups. HCPs and patients referred to the positive effect of PROM in being sensitized for different symptom complexes, reminding them of essential topics during the patient-doctor encounter; this could be especially beneficial for less experienced doctors.

*“For sensitization at the beginning, when I was new here, that was really worth its weight in gold, these sheets. Because otherwise I think that would have slipped through my fingers a bit.”*

*(male doctor 3, second lung cancer focus group)*

#### 3.3.7 Perspectives beyond PROM instruments

The ICGs used the focus group and interview to address also their well-known burden as a caring relative, which may be subject to an assessment of its own. The IPOS psychosocial and spiritual items in particular caught their interest. ICG experience burden by care and by a lack of coordinated care in the hospital.

*“My husband's illness dominates our lives. You don't have any freedom and there is the fact that you often experience thoughtlessness. You are ordered over there and then sent back here again, and so on. That really gets to me.”*

*(wife 2, ICG focus group)*

Also the patients used the focus groups to address issues beyond direct PROM. Of these, we consider distress experienced by potentially avoidable waiting times worthy of attention, because time is a precious commodity in palliative situations.

*“But as I always say: we are all seriously ill patients. The time we have left to live, I don't want to spend most of it in here. And that is the crux. Everything takes time, no question, but a lot of time is wasted here. Our time. My time.”*

*(female patient 1, lung cancer focus group)*

## 4 Discussion

We tested the feasibility of PROM in outpatients with non-curable lung cancers and CRPC. PROM is a prerequisite for being a designated comprehensive cancer center. We used mixed methods integrating the patient perspective, because a comprehensive analysis facilitates implementation and sustainability in the future.

The aim to assess all eligible patients was nearly achieved, but only 50% of the CRPC patients, and 31% of the advanced lung cancer patients were repeatedly (quarterly) assessed as recommended, although patients and health care professionals predominantly found PROM to be useful for the physician-patient encounter. Also, identified distress was hardly ever addressed with referral to supportive and palliative services. Reasons included organizational deficiencies in the handling of the questionnaire and the lack of response operationalization to distress. A small change by monitoring the hand-over of the questionnaires improved their implementation.

### 4.1 Problems of distribution

Although the total rate of screenings by PROM during the study period of 15 months was high, and PROM showed good face validity, the DKG demand to screen all patients was not met. Due to missing routines for IPOS, DT was distributed more often, but also the DT was not as well established as supposed.

One reason may be gate keeping by staff intending to protect patients from supposed burden by PROM. At the beginning of the study when screening rates were low, it was hypothesized that patients might refuse to participate in screenings or feel stressed by them. As also shown in previous studies [18] our patient interviews proved this to be a wrong assumption, but HCP argued PROMs could lead to more distress in patients, which is why some distributed them reluctantly. When PROM distribution was organized and conducted by a researcher in the fifth quarter, screening rates were much higher. Also, the postulated regularity of screenings was not reached for several reasons. Firstly, some patients did not visit the outpatient clinic once every quarter as appointments vary due to therapy plans and the patients' current health. Secondly, staff mentioned they easily forgot about distributing PROMs when they had a stressful day. Others also found that a responsible person to distribute PROMs demonstrably increases screening rates [32]. Management of the immense workload to distribute and interpret the PROM-based screening was a factor recently identified in determining the screening rate in a large multicenter study of the DKG [46].

### 4.2 Problems of consequences

Our study showed that IPOS was more sensitive to detecting distress in patients than DT. The rate of distressed prostate cancer patients was twice as high with IPOS compared to DT. A possible reason could be DT's cut-off of five. In 2005 Jacobson et al. recommended, according to NCCN, a cut-off of four [33,34], which had resulted in 9% more signals of distress in our cohort. Another reason may be that DT posed more problems to patients in terms of understanding how to use the questionnaires. The thermometer was misleading for some patients, which might have been caused by DT's layout and the smaller size of the thermometer compared to the validated original version. Patients were tempted to fill in DT rather thoughtlessly due to only a numerical value and the dichotomous problem list. In contrast, the IPOS Likert-Scale enabled patients to grade and reflect their current situation more precisely, a feedback given in all focus groups.

Also, a response to distress followed an IPOS more often than a DT signalizing distress. Notably, others found that patients rated their burden more severely than HCP [[35], [36], [37]], but we identified physicians reacting to questionnaires indicating no distress. This observation may reflect a more profound change of approach to the patient - rather than the disease - provoked by PROM. Braulke et al. also found referrals of negatively screened patients to supportive and palliative services, that they attributed to uncertainties with cut-off values [46].

We thought that the change process may take time as some physicians pretend not to know about PROM and how to use it. The same effect was seen in the rare referrals to supportive and palliative services, which was perceived as a major omission as patient interviews showed. Noticeable consequences from PROM results are important for patients, as their absence caused frustration and a lack of understanding for the use of PROM. Others also found negative effects for patients when assessments create false expectations, which underlines the importance of enabling a patient's enrollment in supportive and palliative services as well as conducting supportive consultations [38]. Skilled staff and IT solutions are supposed to essentially facilitate targeted consultations and referrals [46].

Only a few patients reported about having used supportive and palliative services. They worked well for patients who had experienced them, their feedback stressing the well-known importance of physicians' referrals [5,17,24].

### 4.3 Strengths and limitations

The generalizability of the study findings is limited to patients with only two different cancer entities and a single center outpatient setting, which is, however, representative for comprehensive cancer centers in Germany.

Reasons for irregularities in patients' visits to the outpatient clinic were only explored for pragmatic reasons, but not systematically assessed.

The unplanned expansion of the study period to a 5th quarter may be considered a methodological flaw. The intention was to provide additional information regarding organizational determinants in PROM distribution, which was eventually attributed to the difference between research and routine PROM processes – findings with far-reaching practical implications for patients when knowledge transfer fails ‘from bench to bedside’ and important palliative needs are not addressed.

A lack of training in palliative care could be one factor that may have contributed to the flaws of the screening. In contrast to the other supportive services palliative care may not have been established in undergraduate studies of all participating HCP. However, it was our omission not to assess vocational training and degrees together with the demographic assessment.

We should have involved patient stakeholders from the planning stage of the study. However, we closely listened to the experience with the particular PROM under real life conditions, and we deliberately chose a mixed methods design to explore the complex nature of needs related to PROM in patients with advanced cancer. Actually, interviews with patients also brought up issues not related to PROM but to distress. Important topics for patients, such as avoidance of long waits, organizational deficiencies, or repeated non-salutary assessment of information about them can be used in subsequent studies and improve advanced cancer outpatients' QoL by reducing nosocomial distress.

### 4.4 Innovation

Exclusive use of quantitative research methods impedes explanation of one of the primary outcomes of palliative care: patients' QoL. Participation in mixed-methods studies where patients are enabled to talk about their problems openly can be a positive experience and help improve well-being [39]. The use of PROM to screen patients for increased distress and monitor their QoL was examined and displayed in detail by using mixed methods [[40], [41], [42]], showing opportunities and constraints in their use in routine clinical care, and generating results for future use when PROM becomes part of patients' treatments. Others have already shown that easy access to PROM results [43] as well as systematic operationalization of PROM consequences are feasible [44]. We found the integration of the patient perspective essentially improving the implementation of PROM under everyday clinical conditions.

### 4.5 Conclusion

PROM with DT and IPOS in clinical routine care show high face validity, do not cause any discomfort for patients, and help HCPs to focus and structure their time for patient visits. IPOS showed a higher degree of content validity and sensitivity for distress in patients compared to DT, especially with CRPC. Low rates of distribution and response to distress are attributed to flawed organizational processes, some HCP gate keeping and implementation challenges that would not be tolerated in other diagnostic and treatment procedures for the benefit of patients. A key finding is that screening under everyday clinical conditions is a complex intervention that requires clear organizational procedures and additional trained staff. Next steps will be testing IPOS assessment for all cancer outpatients, and effective ways of operationalizing reactions to identified needs.

## Funding

This research did not receive any specific grant from funding agencies in the public, commercial, or not-for-profit sectors.

## CRediT authorship contribution statement

**Eileen Ratzel:** Conceptualization, Data curation, Formal analysis, Investigation, Writing – original draft, Writing – review & editing, Visualization. **Ina Maria Pretzell:** Conceptualization, Resources, Writing – review & editing. **Thomas Kindler:** Conceptualization, Resources, Writing – review & editing. **Martin Weber:** Formal analysis, Resources, Software, Supervision, Validation, Writing – review & editing. **Christina Gerlach:** Conceptualization, Formal analysis, Investigation, Methodology, Project administration, Supervision, Writing – original draft, Writing – review & editing.

## Declaration of competing interest

The authors declare the following financial interests/personal relationships which may be considered as potential competing interests:

Christina Gerlach reports a relationship with Amgen that includes: consulting or advisory.

## Appendix A Supplementary data

Supplementary material 1: GRAMMS criteria table Image 1

Supplementary material 2: IPOS, NCCN-DT Image 2

Supplementary material 3: Topic guide Image 3
