# Supplementary material for: Patient Reported Outcome Measurement (PROM) under real-life conditions of non-curable cancer outpatients with the Integrated Palliative Outcome Scale (IPOS) and NCCN-Distress Thermometer – A mixed methods study
Source: PEC Innov. 2024 Feb 12;4:100264. doi: 10.1016/j.pecinn.2024.100264 (PMC10883829; doi:10.1016/j.pecinn.2024.100264)
Supplement: Supplementary file 1 — Supplementary material 1: GRAMMS criteria table [file mmc1.docx]

Good Reporting of A Mixed Methods Study (GRAMMS)

| criterion | Description | page |
| --- | --- | --- |
| (1) Describe the justification for using a mixed methods approach to the research question | We analysed the feasibility of two PROM instruments in patients with advanced lung and prostate cancers in a comprehensive cancer outpatient center. Counting questionnaires, the PROM results and reactions to identified distress must be supplemented by exploring the experience with PROM in all involved in this vulnerable patient group, because a comprehensive analysis facilitates implementation and sustainability in the future. | 1, 11 |
| (2) Describe the design in terms of the purpose, priority and sequence of methods | The distribution, return, and use of PROM results were analyzed with descriptive statistics, and supplemented by focus groups, interviews, and field notes to explore the experience of patients, the informal caregivers, and the health care professionals involved with PROM.  The quantitative, descriptive study phase was accompanied by field notes to inform the interview guide, and followed by the focus groups and interviews. | 3, 5 |
| (3) Describe each method in terms of sampling, data collection and analysis | The comprehensive cancer center daily lists in the clinical information system were used to find out how many eligible patients actually visited the outpatient clinic within 15 months.  *Quantitative Methods*  The DT had already been established for routine clinical assessment, but implementation and results had not been evaluated. Outpatient clinic’s physicians were asked to identify eligible patients and note on their routing slips to provide them with an IPOS in addition to the DT at their next appointment, and the patients’ charts were labeled with a sticker “IPOS”. The distribution of questionnaires was handled by nursing staff at a patient’s first appointment in the respective quarter of the year. The process was supported by a research assistant in the 5^th^ quarter of the study to check for confounders, e.g. gate keeping, time constraints, inadvertent forgetting. Patients filled them in while waiting and were asked to hand them to their attending physicians at consultation.  *Data analysis*  *Statistics*  A binomial test (26) whether the frequency distribution of a dichotomous variable corresponds to an assumed distribution at a significance level of 5% was used to check if the DKG’s specifications for questionnaire distribution (100% of eligible patients received a questionnaire) were met (8).  Independence of variables in fourfold tables was calculated with Pearson’s chi-squared tests, and Fisher’s exact test was computed with figures below five (27), using SPSS 23 to analyze whether a referral to supportive and palliative services or a counselling had taken place following a positive screening result for increased distress, or by chance.  *Qualitative Methods*  Field notes to record observations were constantly taken during the project and informed the interview guide.  Focus group participants, identified by purposive sampling, provided informed consent and were given the option to fill in a demographic data sheet. Focus group interviews with patients, their ICG and HCPs were based on topic guides developed by the authors. The interviews were conducted by C.G., a trained clinical scientist (M.Sc.) and physician, audio-recorded and transcribed verbatim (anonymized). The transcripts were thematically analyzed using the framework approach (28, 29), which is based on a matrix that helps to inductively organize the text and identify thematic categories from codes within and across the focus groups. The coders had different levels of prior experience, the first author E.R. was an undergraduate medical student pursuing her doctoral thesis and supported by C.G. Meaning sections were coded without counts of frequencies. Categories were identified from codes and condensed following iterative consultation and agreement of the coders. A data matrix generated on the basis of identified categories and corresponding quotes was used as a template for the framework. Categories were further analyzed, emphasizing similarities and differences. In conclusion, comparisons were made, hypothesized, and explanations drafted. | 3, 5 |
| (4) Describe where integration has occurred, how it has occurred and who has participated in it | The study started with the quantitative part, distribution of questionnaires (outpatient clinic staff, I.P.) and its observation taking field notes (C.G., E.R.), which informed the interview guide (C.G., E.R.) for the focus groups. Results from the focus groups were taken forward to the last three months of the observed PROM distribution, when a research assistant supported the distribution to control gate keeping and organizational deficits (E.R.). |  |
| (5) Describe any limitation of one method associated with the present of the other method | The methods complemented and enriched each other. | - |
| (6) Describe any insights gained from mixing or integrating methods | Due to deficiencies in the organizational handling of the instruments (field notes, focus groups) and the lack of operationalization of reactions to identified distress (focus groups), response rates and the number of referrals to supportive and palliative services were low (quantitative).  Our purpose for a comprehensive analysis to facilitate implementation and sustainability of PROM in vulnerable patients in the future was accomplished, because next steps will be testing IPOS assessment for all outpatients, and effective ways of operationalizing reactions to identified needs. | 1, 11, 13 |

O’Cathain A, Murphy E, Nicholl J. The Quality of Mixed Methods Studies in Health Services Research. *Journal of Health Services Research & Policy*. 2008;13(2):92-98. doi:[10.1258/jhsrp.2007.007074](https://doi.org/10.1258/jhsrp.2007.007074)
