# Supplementary material for: Patient Reported Outcome Measurement (PROM) under real-life conditions of non-curable cancer outpatients with the Integrated Palliative Outcome Scale (IPOS) and NCCN-Distress Thermometer – A mixed methods study
Source: PEC Innov. 2024 Feb 12;4:100264. doi: 10.1016/j.pecinn.2024.100264 (PMC10883829; doi:10.1016/j.pecinn.2024.100264)
Supplement: Supplementary file 2 — Supplementary material 2: IPOS, NCCN-DT [file mmc2.pdf]

Kleiner Aufkleber

IPOS Patienten Version

Name:

Datum:..

Bitte beantworten Sie die folgenden Fragen. Durch Ihre Antworten helfen Sie uns, Ihre Betreuung zu verbessern. Herzlichen Dank.

1. Was bereitet Ihnen zurzeit die größten Probleme oder Sorgen?

2. Unten finden Sie eine Liste mit Symptomen, die Sie unter Umständen haben. Bitte kreuzen Sie an, wie sehr diese Symptome Sie in der letzten Woche *beeinträchtigt* haben.

	Gar nicht	*Ein wenig*	*Mäßig*	*Stark*	*Extrem stark*
**Schmerzen**	0☐	1☐	2☐	3☐	4☐
**Atemnot**	0☐	1☐	2☐	3☐	4☐
**Schwäche oder fehlende Energie**	0☐	1☐	2☐	3☐	4☐
**Übelkeit** *(das Gefühl erbrechen zu müssen)*	0☐	1☐	2☐	3☐	4☐
**Erbrechen**	0☐	1☐	2☐	3☐	4☐
**Appetitlosigkeit**	0☐	1☐	2☐	3☐	4☐
**Verstopfung**	0☐	1☐	2☐	3☐	4☐
**Mundtrockenheit oder schmerzhafter Mund**	0☐	1☐	2☐	3☐	4☐
**Schläfrigkeit**	0☐	1☐	2☐	3☐	4☐
**Eingeschränkte Mobilität**	0☐	1☐	2☐	3☐	4☐

Bitte nennen Sie weitere Symptome, die Sie in der letzten Woche *beeinträchtigt* haben.

**1.**	0☐	1☐	2☐	3☐	4☐
**2.**	0☐	1☐	2☐	3☐	4☐
**3.**	0☐	1☐	2☐	3☐	4☐

In der letzten Woche:

	Gar nicht	*Selten*	*Manchmal*	*Meistens*	*Immer*
**3. Waren Sie wegen Ihrer Erkrankung oder Behandlung besorgt oder beunruhigt?**	0☐	1☐	2☐	3☐	4☐
**4. Waren Ihre Familie oder Freunde Ihretwegen besorgt oder beunruhigt?**	0☐	1☐	2☐	3☐	4☐
**5. Waren Sie traurig bedrückt?**	0☐	1☐	2☐	3☐	4☐

	Immer	*Meistens*	*Manchmal*	*Selten*	*Gar nicht*
**6. Waren Sie im Frieden mit sich selbst?**	0☐	1☐	2☐	3☐	4☐
**7. Konnten Sie Ihre Gefühle mit Ihrer Familie oder Ihren Freunden teilen, so viel wie Sie wollten?**	0☐	1☐	2☐	3☐	4☐
**8. Haben Sie so viele Informationen erhalten, wie Sie wollten?**	0☐	1☐	2☐	3☐	4☐

	Probleme angegangen Keine Probleme	*Probleme größtenteils angegangen*	*Probleme teilweise angegangen*	*Probleme kaum angegangen*	*Probleme nicht angegangen*
**9. Wurden praktische Probleme angegangen, die Folge Ihrer Erkrankung sind (z.B. finanzieller oder persönlicher Art)?**	0☐	1☐	2☐	3☐	4☐

	Ich alleine	*Mit der Hilfe eines Angehörigen oder Freundes*	*Mit Hilfe eines Mit-arbeiters*
**10. Wie haben Sie den Fragebogen ausgefüllt?**	☐	☐	☐

Wenn Sie über eine der Fragen beunruhigt sind, sprechen Sie bitte mit Ihrem Arzt/ Ihrer Ärztin oder Ihrer Pflegekraft.

Handzeichen Arzt: ____________________ Vom Arzt auszufüllen: ◯ Problem im persönlichen Gespräch mit Patient:in angegangen
◯ Konsil Sozialdienst ◯ Schmerzkonsil ◯ Palliativkonsil ◯ Psychoonkologie ◯ Physiotherapie ◯ Ernährungsteam angemeldet

UNIVERSITÄTSmedizin.
uct | Universitäres Centrum für Tumorerkrankungen MAINZ
Sektion Psychoonkologie (SPO)
Klinik für Psychosomatische Medizin und Psychotherapie

Fragebogen zu Belastungen

(V.1.2016, es gilt die aktuelle elektronische Version)

Datum:

Pat.-Aufkleber

Bitte kreuzen Sie auf der nebenstehenden Skala mit einem **X** eine Zahl von **0** bis **10** an.
Diese Zahl zeigt an, wie stark Sie Ihre Belastungen in der letzten Woche einschließlich des heutigen Tages empfinden.

Stärkste Belastung

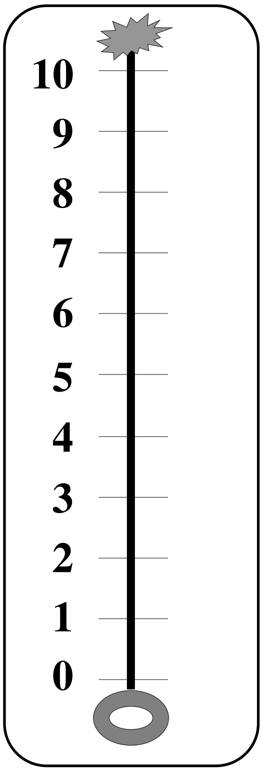

Keine Belastung

Kreuzen Sie bitte unten an, was für Sie in der letzten Woche einschließlich des heutigen Tages belastend gewesen ist.
Bitte vergewissern Sie sich, dass Sie alle Punkte mit **JA** oder **NEIN** beantwortet haben:

JA	NEIN	**Praktische Probleme**
☐	☐	Kinderbetreuung
☐	☐	Wohnsituation
☐	☐	Versicherung/Finanzen
☐	☐	Mobilität/Beförderung
☐	☐	Arbeit/Ausbildung
		**Familie**
☐	☐	Umgang mit Kindern
☐	☐	Umgang mit Partner
☐	☐	Umgang mit Eltern
		**Emotionale Probleme**
☐	☐	Depressivität
☐	☐	Angst
☐	☐	Nervosität
☐	☐	Traurigkeit
☐	☐	Sorgen
☐	☐	Interesselosigkeit für alltägliche Aktivitäten
☐	☐	**Spirituelle/religiöse Angelegenheiten**

JA	NEIN	**Körperliche Probleme**
☐	☐	Aussehen
☐	☐	Körperpflege/Ankleiden
☐	☐	Atmung
☐	☐	Wasserlassen
☐	☐	Verstopfung
☐	☐	Durchfall
☐	☐	Appetit
☐	☐	Müdigkeit
☐	☐	Ödeme/Stauungen
☐	☐	Fieber
☐	☐	alltägliche Verrichtungen
☐	☐	Verdauung
☐	☐	Gedächtnis/Konzentration
☐	☐	Mundschleimhaut
☐	☐	Übelkeit
☐	☐	trockene/verstopfte Nase
☐	☐	Schmerz
☐	☐	Sexualität
☐	☐	trockene/juckende Haut
☐	☐	Schlaf
☐	☐	Kribbeln in Händen/Füßen

Weitere Probleme:

Gesehen (Arzt/Ärztin):

Konsil: ☐ ja / ☐ nein
