# Supplementary material for: Patient Reported Outcome Measurement (PROM) under real-life conditions of non-curable cancer outpatients with the Integrated Palliative Outcome Scale (IPOS) and NCCN-Distress Thermometer – A mixed methods study
Source: PEC Innov. 2024 Feb 12;4:100264. doi: 10.1016/j.pecinn.2024.100264 (PMC10883829; doi:10.1016/j.pecinn.2024.100264)
Supplement: Supplementary file 3 — Supplementary material 3: Topic guide [file mmc3.pdf]

Topic Guide

P A T I E N T E N

Studie zur Machbarkeit einer wiederholten strukturierten Erhebung der Lebensqualität als Indikator für Versorgungsbedarf bei Patienten mit kastrationsrefraktärem Prostatakarzinom sowie bei Patientinnen und Patienten mit nicht-heilbarem Lungenkrebs – qualitative Exploration (II)

POSPROS

Zielsetzung:

- Exploration der persönlichen Erfahrungen der Patienten beim Ausfüllen des IPOS-Fragebogens und des Fragebogens zur Belastung (Distressthermometer)

- *<u>Einführung:</u>*

  - **Begrüßung:** Vielen herzlichen Dank, dass Sie sich die Zeit nehmen, um sich an der Besprechung zu den IPOS-Fragebögen und Distressthermometer zu beteiligen.

  - **Vorstellung Christina/ Vorstellung Eileen:** Ich bin Christina Gerlach und arbeite als Ärztin hier im Haus, im Moment schwerpunktmäßig in der Forschung. Mein Name ist Eileen Ratzel, ich schreibe meine Doktorarbeit über die Verwendung der Fragebögen auf der uct-Ambulanz und deren Auswirkungen auf die Behandlung der Patienten. Was möchten sie noch gerne über uns wissen?

  - **Studienzweck:** Wir haben diese Fragebögen an Sie verteilt, um festzustellen, ob es möglich ist, damit frühzeitig auf Probleme von Patienten mit fortgeschrittenen Krebserkrankungen aufmerksam zu werden, und ob und wie die Ärzte auf Signale aus den Fragebögen reagieren. Da die Deutsche Krebsgesellschaft solche Überprüfungen der Belastung der Patienten vorsieht, wollten wir zusätzlich herausfinden, ob eine solche Art der Befragung überhaupt auf der uct-Ambulanz möglich ist und wie sie optimiert werden kann, um auf die Bedürfnisse der Patienten eingehen zu können.

- **Vertraulichkeit:** Alles, was Sie hier sagen, wird vertraulich behandelt. Wir unterliegen der Schweigepflicht. Wir zeichnen das Gespräch auf, allerdings notieren wir dazu keinerlei persönliche Daten. So bleiben ihre Aussagen anonym. Sie haben das Recht, die Befragung jederzeit zu unterbrechen oder abzubrechen. Sie dürfen alles sagen, was Ihnen zu unseren Fragen einfällt, gerne auch etwas ergänzen, das Ihnen zum Thema auf dem Herzen liegt. Wir sind an all Ihren Gedanken und Ansichten interessiert.
- Haben Sie noch Fragen zur Durchführung? Wenn nicht und Sie sich bereit fühlen, beginnen wir nun mit der Aufnahme und dem Interview.

--AUFNAHME STARTEN--

<u>Organisation</u>

- Welche Fragebögen haben Sie bei Ihren Besuchen auf der uct-Ambulanz erhalten?

- Wurde Ihnen erklärt, weshalb Sie den FB ausfüllen sollen?

- Wie viel Zeit haben Sie in etwa für das Ausfüllen der FB benötigt?

- Wo haben Sie die FB ausgefüllt?

<u>Meinung</u>

- Weshalb haben Sie den Fragebogen ausgefüllt?

- Was halten Sie vom DT? Was halten Sie vom IPOS?

- Welchen der beiden Fragebögen bevorzugen Sie?

    Warum?

Individuelle Erfahrungen

- Wie haben Sie das Ausfüllen des FB empfunden?

    Ausfüllen im Wartezimmer neben anderen Pat. = Problem?

- Was glauben Sie, was mit den ausgefüllten Fragebögen geschieht?

- Welche Auswirkungen hatte der FB für Sie?

    Welche persönlichen Vor- oder Nachteile haben sich für Sie durch das Ausfüllen der FB ergeben?

- Würden Sie anderen Patienten empfehlen, FB auszufüllen?

- Inwieweit hat Sie das Ausfüllen der Fragebögen belastet?

    Wurden Ihnen durch das Ausfüllen der FB eigene Probleme bewusster?

- Welche Unterstützung wurde Ihnen von Ihrer Ärztin/ Ihrem Arzt angeboten, wenn Sie etwas belastet hat?

Fragen zur Krebserkrankung

- Wie sehr sind Sie in ihrem Alltag durch Ihre Erkrankung eingeschränkt?

- Welche Auswirkungen hat Ihre Erkrankung auf Ihr Privatleben?

Abschließende Frage

Was möchten Sie gerne noch ergänzen? Was ist Ihnen noch wichtig, wonach noch nicht gefragt wurde?

--AUFNAHME ANHALTEN--

-----------------------------HERZLICHEN DANK---

---DEBRIEFING --

- Wie geht es Ihnen im Moment? Was können wir im Moment für Sie tun?
- Haben Sie nochmals vielen Dank für Ihre Teilnahme, Sie helfen uns sehr weiter.
- Falls Sie noch Fragen haben, können Sie uns jederzeit kontaktieren. Sie finden die Kontaktdaten auf dem Informationsschreiben und wir haben hier nochmal Kärtchen *(Aushändigen von Kärtchen mit Kontaktdaten).*

Topic Guide

Medizinisches Personal

Studie zur Machbarkeit einer wiederholten strukturierten Erhebung der Lebensqualität als Indikator für Versorgungsbedarf bei Patienten mit kastrationsrefraktärem Prostatakarzinom sowie bei Patientinnen und Patienten mit nicht-heilbarem Lungenkrebs – qualitative Exploration (II)

POSPROS

Zielsetzung:

- Exploration der persönlichen Erfahrungen von Mitarbeiterinnen und Mitarbeitern der uct-Ambulanz mit dem IPOS-Fragebogen und dem Fragebogen zur Belastung (Distressthermometer)
- Durchführbarkeit, Einbringung in den Arbeitsalltag

- *Einführung:*

  - **Begrüßung:** Vielen herzlichen Dank, dass Sie sich die Zeit nehmen, um sich an der Besprechung zu den IPOS-Fragebögen und Distressthermometer zu beteiligen.

  - **Vorstellung Eileen:** Mein Name ist Eileen Ratzel, Sie haben mich während der Studie bereits kennengelernt. Vielen Dank, dass Sie mich hiermit nun noch weiter unterstützen.

  - **Studienzweck:** Die Fragebögen werden verteilt, um festzustellen, ob es mit Ihnen im Praxisalltag möglich ist, besser auf Probleme von Patienten mit fortgeschrittenen Krebserkrankungen aufmerksam zu werden. Die Deutsche Krebsgesellschaft macht solche Überprüfungen der Belastung der Patienten zur Vorgabe für die Zertifizierung, und wir wollten zusätzlich herausfinden, ob eine solche Art der Befragung überhaupt auf der uct-Ambulanz möglich ist und wie sie optimiert werden kann, um möglichst frühzeitig auf die Bedürfnisse der Patienten eingehen zu können.

- **Vertraulichkeit:** Alles, was Sie hier sagen, wird vertraulich behandelt. Wir unterliegen der Schweigepflicht. Wir zeichnen das Gespräch auf, allerdings notieren wir dazu keinerlei persönliche Daten. So bleiben ihre Aussagen anonym. Sie haben das Recht, die Befragung jederzeit zu unterbrechen oder abzubrechen. Sie dürfen alles sagen, was Ihnen zu unseren Fragen einfällt, gerne auch etwas ergänzen, das Ihnen zum Thema auf dem Herzen liegt. Wir sind an all Ihren Gedanken und Ansichten interessiert.
- Haben Sie noch Fragen zur Durchführung? Wenn nicht und Sie sich bereit fühlen, beginnen wir nun mit der Aufnahme und dem Interview.

--AUFNAHME STARTEN--

Organisation

- Welche Fragebögen haben die Patienten ihrer Erfahrung nach erhalten?

- Welche Fragebögen haben die Patienten zur Sprechstunde mitgebracht?

- Welche Fragen hatten die Patienten zum Ausfüllen des Fragebogens?

- Wie viel Zeit benötigten Sie zur Besprechung des Fragebogens?

    Welchen Anteil hat die Besprechung des Fragebogens an ihrem Gespräch eingenommen?

Meinung

- Was halten Sie vom DT? Was halten Sie vom IPOS?

- Welchen der beiden Fragebögen bevorzugen Sie?

    Welche Vor- und Nachteile haben die Fragebögen?

Individuelle Erfahrungen

- Welchen Einfluss hatte der Fragebogen auf Ihre Arbeit mit den Patienten?

  Welche Auswirkungen hatten die Angaben des Patienten im Fragebogen für Ihre Behandlung?

- Würden Sie weiterhin mit den Fragebögen arbeiten?

- Wie empfanden die Patienten ihrer Meinung nach das Ausfüllen der Fragebögen?

  Welche Arbeitserleichterungen und welche Probleme ergeben sich durch das Ausfüllen der Fragebögen für Sie und für die Patienten?

Abschließende Frage

Was möchten Sie gerne noch ergänzen? Was ist Ihnen noch wichtig, wonach noch nicht gefragt wurde?

---------------------------------------AUFNAHME ANHALTEN--

---------------------------HERZLICHEN DANK FRAU/HERR ………….---------------------------------------

--DEBRIEFING ---

- Wie geht es Ihnen im Moment? Was können wir im Moment für Sie tun?
- Haben Sie nochmals vielen Dank für Ihre Teilnahme, Sie helfen uns sehr weiter.
- Falls Sie noch Fragen haben, können Sie uns jederzeit kontaktieren.

Topic Guide

A N G E H Ö R I G E

Studie zur Machbarkeit einer wiederholten strukturierten Erhebung der Lebensqualität als Indikator für Versorgungsbedarf bei Patienten mit kastrationsrefraktärem Prostatakarzinom sowie bei Patientinnen und Patienten mit nicht-heilbarem Lungenkrebs – qualitative Exploration (II)

POSPROS

Zielsetzung:

- Exploration der persönlichen Erfahrungen der Angehörigen bzw. Begleitpersonen von Patienten mit fortgeschrittenem Lungen- und kastrationsrefraktärem Prostatakarzinom, die bei ihren Besuchen auf der uct-Ambulanz den IPOS-Fragebogen und den Fragebogen zur Belastung (Distressthermometer) ausgefüllt haben

---

- *Einführung:*

  - **Begrüßung:** Vielen herzlichen Dank, dass Sie sich die Zeit nehmen, um sich an der Besprechung zu den IPOS-Fragebögen und Distressthermometer zu beteiligen.

  - **Vorstellung Christina/ Vorstellung Eileen:** Ich bin Christina Gerlach und arbeite als Ärztin hier im Haus, im Moment schwerpunktmäßig in der Forschung. Mein Name ist Eileen Ratzel, ich schreibe meine Doktorarbeit über die Verwendung der Fragebögen auf der uct-Ambulanz und deren Auswirkungen auf die Behandlung der Patienten. Was möchten Sie noch gerne über uns wissen?

- **Studienzweck:** Wir haben diese Fragebögen an Ihre Angehörigen verteilt, die hier Patienten in der uct-Ambulanz sind, um festzustellen, ob es möglich ist, damit frühzeitig auf Probleme von Patienten mit fortgeschrittenen Krebserkrankungen aufmerksam zu werden, und ob und wie die Ärzte auf Signale aus den Fragebögen reagieren. Da die Deutsche Krebsgesellschaft solche Überprüfungen der Belastung der Patienten vorsieht, wollten wir zusätzlich herausfinden, ob eine solche Art der Befragung überhaupt auf der uct-Ambulanz möglich ist und wie sie optimiert werden kann, um auf die Bedürfnisse der Patienten eingehen zu können. Bei einer schweren Erkrankung geht es aber nicht nur dem Patienten oder der Patientin selbst schlechter, sondern auch denen, die ihnen nahestehen. Deswegen ist es für uns sehr wichtig, Ihre Meinung zum Thema Fragebögen und den damit zusammenhängenden Abläufen zu erfahren.

- **Vertraulichkeit:** Alles, was Sie hier sagen, wird vertraulich behandelt. Wir unterliegen der Schweigepflicht. Wir zeichnen das Gespräch auf, allerdings notieren wir dazu keinerlei persönliche Daten. So bleiben Ihre Aussagen anonym. Sie haben das Recht, die Befragung jederzeit zu unterbrechen oder abzubrechen. Sie dürfen alles sagen, was Ihnen zu unseren Fragen einfällt, gerne auch etwas ergänzen, das Ihnen zum Thema auf dem Herzen liegt. Wir sind an all Ihren Gedanken und Ansichten interessiert.
- Haben Sie noch Fragen zur Durchführung? Wenn nicht und Sie sich bereit fühlen, beginnen wir nun mit der Aufnahme und dem Interview.

--AUFNAHME STARTEN--

Organisation

- Welche Rolle haben Sie in der Betreuung/Begleitung ihres Angehörigen während der

Behandlung auf der uct-Ambulanz eingenommen?

- Welche Fragebögen hat Ihr Angehöriger auf der uct-Ambulanz ausgefüllt?

    Wie haben Sie von den FB erfahren?

Meinung

- Was halten Sie von den FB?

    Würden Sie als Patient FB ausfüllen?

    Welchen FB würden Sie bevorzugen?

- Welche Vor- und Nachteile sehen Sie in der Befragung der Patienten durch FB?

Individuelle Erfahrungen

- Wie wurden Sie in die Behandlung Ihres Angehörigen miteinbezogen?

    Wie wurde auf Ihre Sorgen und Ängste eingegangen?

- Welche Konsequenzen haben sich für Ihren Angehörigen und Sie aus dem Ausfüllen der FB ergeben?

    Welche persönlichen Vorteile hat Ihr Angehöriger durch das Ausfüllen der FB erfahren?

    Welche Belastungen und Probleme ergaben sich durch das Ausfüllen der FB?

Privatleben

- Wie hat sich Ihr Leben verändert, seitdem Ihr Angehöriger die Diagnose erhalten hat?

    Wie sehr sind Sie durch die Erkrankung Ihres Angehörigen belastet?

Abschließende Frage

Was möchten Sie gerne noch ergänzen? Ist Ihnen noch etwas wichtig, wonach noch nicht gefragt wurde?

--AUFNAHME ANHALTEN---

----------------------------HERZLICHEN DANK--

---DEBRIEFING --

- Wie geht es Ihnen im Moment? Was können wir im Moment für Sie tun?
- Haben Sie nochmals vielen Dank für Ihre Teilnahme, sie helfen uns sehr weiter.
- Falls Sie noch Fragen haben, können Sie uns jederzeit kontaktieren. Sie finden die Kontaktdaten auf dem Informationsschreiben und wir haben hier nochmal Kärtchen *(Aushändigen von Kärtchen mit Kontaktdaten).*
